# Response to: ‘Use of portable air purifiers to reduce aerosols in hospital settings and cut down the clinical backlog’

**DOI:** 10.1017/S0950268823000547

**Published:** 2023-04-17

**Authors:** Jacob Salmonsmith, Andrea Ducci, Ramanarayanan Balachandran, Liwei Guo, Ryo Torii, Catherine Houlihan, Ruth Epstein, John Rubin, Manish K. Tiwari, Laurence B. Lovat

**Affiliations:** 1 Department of Mechanical Engineering, University College London, London, UK; 2 Rare and Imported Pathogens Laboratory, UK Health Security Agency, London, UK; 3 University College London, London, UK; 4 Department of Otolaryngology, Royal National Ear Nose and Throat and Eastman Dental Hospital, University College London Hospitals NHS Foundation Trust, London, UK; 5 Division of Surgery & Interventional Science, University College London, London, UK; 6 Wellcome/EPSRC Centre for Interventional & Surgical Sciences (WEISS), University College London, London, UK; 7 Gastrointestinal Services, University College London Hospital, London, UK

## Response

We thank Malek, Al-Shehab, and Lo for their kind words in their correspondence regarding our article, ‘*Use of portable air purifiers to reduce aerosols in hospital settings and cut down the clinical backlog’.* Alongside a summary of the paper’s findings, they gave some commentary on the work and suggested some future work.

The first comment made was regarding the lack of masks in the study, and whether the resultant findings may overstate the effectiveness of Portable Air Cleaners (PAC) in settings where masks are worn. We agree with and acknowledge the findings from previous studies that masks can reduce the aerosol spread by up to 60%. However, even in these settings, aerosol transmission of airborne viruses is still possible. The Validation of Experimental Protocol section of the main article and the corresponding section in the Supplementary Information of our paper talk about studies where the aerosol concentration generated by our android was reduced, to see if the decay rate and half-life of the aerosol concentration were affected by using lower filling steady-state aerosol concentrations (FSSACs), to increase confidence that our study bore relevance to human levels of aerosol concentration. We found that the resultant decay rates and half-lives were slightly conservative at higher FSSACs, but the results across various FSSACs showed a clear distinction between the various mitigations. It can reasonably be assumed that further lowering the aerosol concentration due to the use of masks would still result in a distinction between the mitigations, that is, indicating that using a PAC would reduce the risk of infection in an indoor setting where masks were being worn. We also agree that investigating this assumption would be an interesting and useful piece of research.

The second comment suggested using multiple streams from nebulisers, in order to simulate a larger population of people in a setting, with the various resultant air streams from non-infectious sources affecting the infectious stream of aerosols in unpredictable ways. This research would be interesting to undertake, and we agree that the paths of aerosols after being exhaled are complicated and unpredictable due to many factors. For the results presented in the article, we were primarily concerned with the air cleaning rates after the infectious source had left the room, and the aerosol generator was only turned off once we had reached FSSACs, which can be associated with a well-mixed environment. In addition to the previously referenced study, where the mitigations had similar effects on different concentrations of aerosols, this gives us confidence that although the overall aerosol concentration levels of infectious particles within a room may be difficult to predict, due in part to the factors stated by Lo et al., the use of PACs would reduce the levels of these infectious aerosols, especially after the infectious aerosol source had left the room.

The third and final comment suggested that infectious particles may spread in a room of multiple people before any PACs also located there have been able to effectively remove the particles. The use of multiple aerosol/particle detectors would improve the fidelity of the room analysis, especially during seeding, as Lo et al. suggest. The presented work was concerned with the clearing of potentially infectious particles from a consulting room after the occupants have left, in order to make the environment safer for the next occupants. We are currently investigating the spread of aerosols during seeding, and these suggestions are useful in improving the setup and analysis of the experiments.

